# The Effect of Training Exercise on Cartilage Zonal Composition and Structure at Two Differently Loaded Sites in the Juvenile Equine Metacarpophalangeal Joint

**DOI:** 10.1177/19476035261484024

**Published:** 2026-10-11

**Authors:** Michael J. S. Duggan, Margot C. Labberté, René van Weeren, Reinout Stoop, Mika M. Hyttinen, Jarno Rieppo, Jeroen de Groot, Pieter. A. J. Brama

**Affiliations:** 1 Translational Research Unit, School of Veterinary Medicine, College of Health & Agricultural Sciences, University College Dublin, Ireland; 2 UCD Conway Institute of Biomolecular and Biomedical Research, University College Dublin, Belfield, Ireland; 3 Department of Clinical Sciences, Faculty of Veterinary Medicine, Utrecht University, Netherlands; 4 Department of Biomedical Research, 2859 The Netherlands Organization for Applied Scientific Research (TNO), Leiden, The Netherlands; 5 Institute of Biomedicine, Department of Anatomy, 4344 University of Eastern Finland, Kuopio, Finland; 6 Ncardia Services B.V, Leiden, Netherlands

**Keywords:** rticular cartilage, zonal distribution, topographical distribution, cartilage composition, cartilage structure, training exercise, horse

## Abstract

**Background:**

The aim of this study was to investigate the depth related spatial distribution of biochemical and structural components in articular cartilage at different topographical sites experiencing different loading within the juvenile equine metacarpophalangeal joint in two groups of foals subjected to different exercise conditions.

**Methods:**

Sixteen Warmblood foals were included in the study and were randomly assigned into 1 of 2 exercise groups (box rest and box rest plus training) during the first 5 months of life and subsequently euthanized. Osteochondral plugs were harvested from two sites which have previously been shown to experience different load levels and sectioned into 100 μm thick slices. Cartilage slices were analysed for glycosaminoglycan, DNA, and total collagen content, as well as content of hydroxylysine residues (HYL) in collagen and the collagen cross-link Hydroxylysylpyridinoline (HP). Collagen fibril architecture (Orientation Angle and Parallelism Index) was determined using quantitative polarized light microscopy.

**Results:**

The constantly loaded site 2 exhibited higher glycosaminoglycan (GAG) and HP content with HYL higher in the superficial and middle layers of the trained group, while the intermittently peak loaded site 1 showed greater collagen accumulation in deeper layers but lower cross-link density. Structural analyses (Oa and PI) confirmed that collagen fibril orientation and alignment adapt regionally, with training enhancing fibril organization in superficial zones.

**Conclusion:**

This study illustrates that mechanical loading is a key driver of functional adaptation, resulting in distinct zonal and topographical differences in the biochemical and structural composition of equine cartilage at 5 months.

## 1. Introduction

Articular hyaline cartilage covers the surfaces of bones within synovial joints, providing a smooth, low-friction, and wear-resistant surface essential for load distribution in diarthrodial joint function. Despite its simple appearance, articular cartilage possesses a complex structure with multifaceted interactions between the chondrocytes and the extracellular matrix (ECM).^1^ The ECM is composed predominantly of collagen, proteoglycans and water. Articular cartilage is arranged into four zones with variations in density and arrangement of key structural elements.^2-5^ These zones are referred to as; superficial, transitional (middle), deep (radial) and calcified.^3,5^ It is this complex composition and architecture that affords articular cartilage its functional properties.

The superficial zone constitutes the upper 10–20% of the cartilage thickness, within which, fine collagen fibrils are aligned parallel to the articular surface and densely packed.^4,6,7^ Chondrocytes in this portion of the cartilage are typically abundant and flat in shape.^8^ The superficial zone has the highest water and lowest proteoglycan content which, combined with the low collagen fibril constraint, play a key role in the zone’s swelling potential.^3,9^ The middle zone makes up 40–60% of the total thickness with thicker collagen fibres that are generally arranged in a random pattern; however, a tendency to orient at 45° has been reported.^10^ The chondrocytes located in the middle zone are significantly greater in volume and surface area compared with those in the superficial zone^8^ but are less densely packed. Proteoglycan content tends to increase through the middle zone with some reports suggesting it has the highest level of proteoglycans^11,12^ whilst others suggest the highest content is within the deep zone.^13,14^ The deep zone represents 30% of the cartilage thickness; here collagen fibres interweave to form large bundles oriented perpendicularly to the surface, and penetrating the tidemark into the deeper calcified region. These perpendicular fibres provide anchorage to the subchondral bone^15^ and, despite the osmotic potential provided by the proteoglycan content,^16^ the high collagen constraint restricts the swelling potential and subsequently results in a lower water content. In general, the collagen fibril diameter increases from superficial to deep whilst the inverse is true for cell density^8,17^ and water, which tend to decrease.

It is well recognised that loading is a critically important aspect of cartilage development, driving the development of topographical heterogeneity.^18-23^ The aim of this study was to investigate the depth related spatial distribution of biochemical and structural components in articular cartilage at two separate locations within the equine metacarpophalangeal joint which have previously been shown to experience different load levels associated with constant weight bearing and intermittent peak loading during exercise^24^ under different exercise conditions (box rest and box rest plus training).

## 2. Materials & Methods

### 2.1. Study Design

Sixteen Warmblood foals (8 male, 8 female) were included in the study (Figure 1). The foals were allowed paddock turnout with the mares from birth until one week of age. After which, they were randomly assigned into 1 of 2 groups (box, trained). The box group were kept with the mare in a stable of 3m x 3.5 m for 24 h per day. The trained group were stabled in the same sized stables and were subjected to a predefined training programme. The training program is described in detail by Van Weeren et al.,^25^ but briefly, consisted of an increasing number of gallop sprints in a 48 x 15 m paddock constructed with a concrete floor overlayed with a thick layer of sand. At day 7, the program involved 12 sprints, from days 8-24 16 sprints, from days 25-38 24 sprints, and from day 39 to the end of the study, on alternating days, 32 and 16 sprints. Training took place 6 days a week. The foals were subjected to euthanasia using intravenous administration of pentobarbital at 5 months of age. The samples were collected as part of a large project that was approved by the ethics committee of the University of Utrecht.^25^

**Figure 1.**
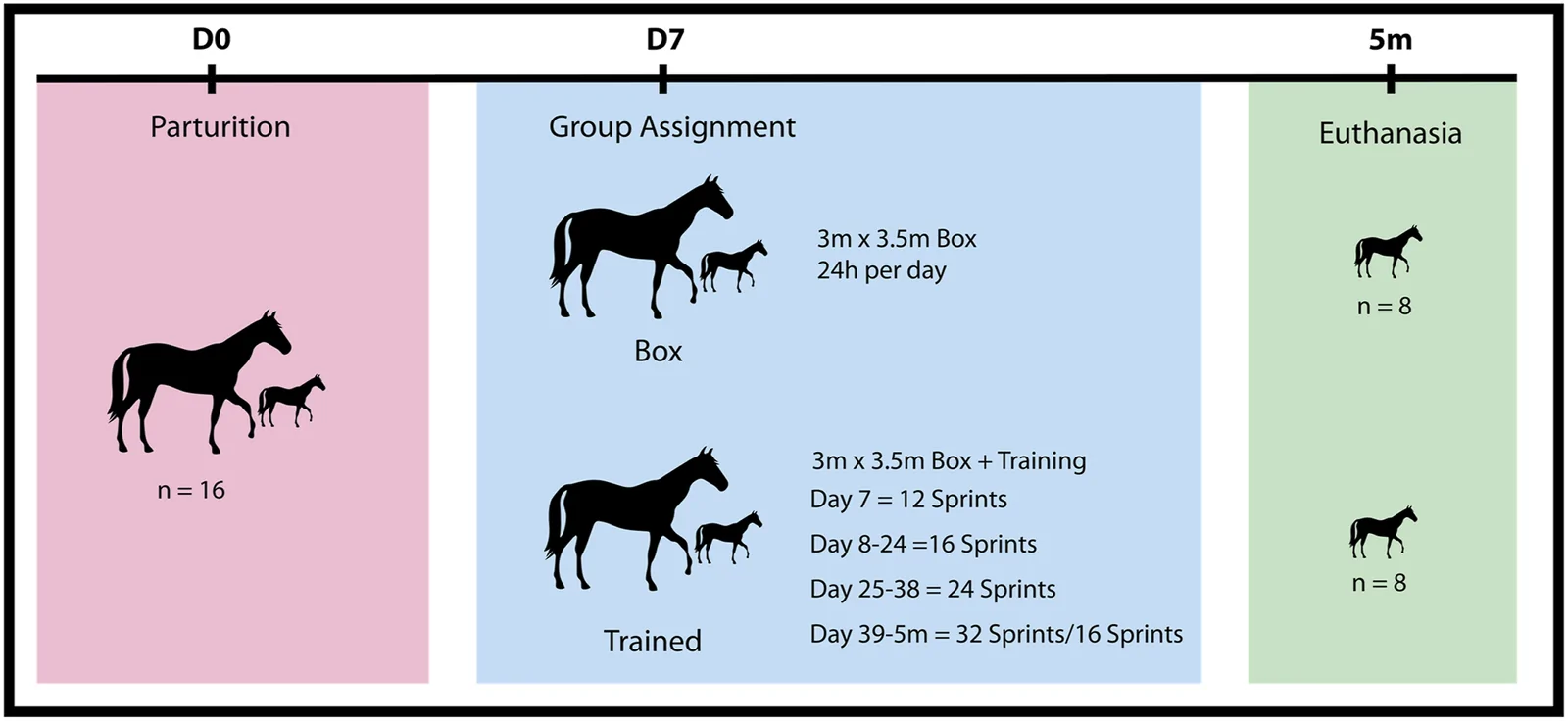
Graphical summary of the study timeline from 0 to 5 months and respective grouping regimens (box, training)

### 2.2. Sample Collection

The left metacarpophalangeal (MCP) joint was removed *en bloc* by transection through the proximal phalangeal bone and distal 3^rd^ metacarpal bone less than 2 hours after euthanasia. The joint was wrapped immediately in cling film and stored at -20°C. After thawing, osteochondral plugs (6 mm ∅) were extracted perpendicular to the articular surface from 2 predefined locations on the lateral side of the proximal articular surface of the proximal phalanx, using a custom-built hollow drill.^26^ One plug was taken from the dorsal edge of this lateral side (Site 1, Figure 2) representing cartilage exposed to peak loading during exercise. The second was harvested from the mid-region of the joint cavity (Site 2, Figure 2) representing constant weight bearing load.^27^ The osteochondral plugs were sectioned into 2 equal halves perpendicular (a, Figure 2) to the articular surface using a 24mm diameter, 0.1mm thick circular blade. One of the halves was subsequently split in half again (b & c, Figure 2). One of the “quarters” (c, Figure 2) was sectioned using a microcryotome into 100 μm slices from superficial to deep and one “quarter” (b, Figure 2) was used as a full thickness cartilage specimen stored at -20^o^C until further biochemical analysis. Half “a”(Figure 2) was demineralised, paraffin embedded and sliced for polarised light microscopic structural analyses. All samples were subsequently analysed by a blinded operator.

**Figure 2.**
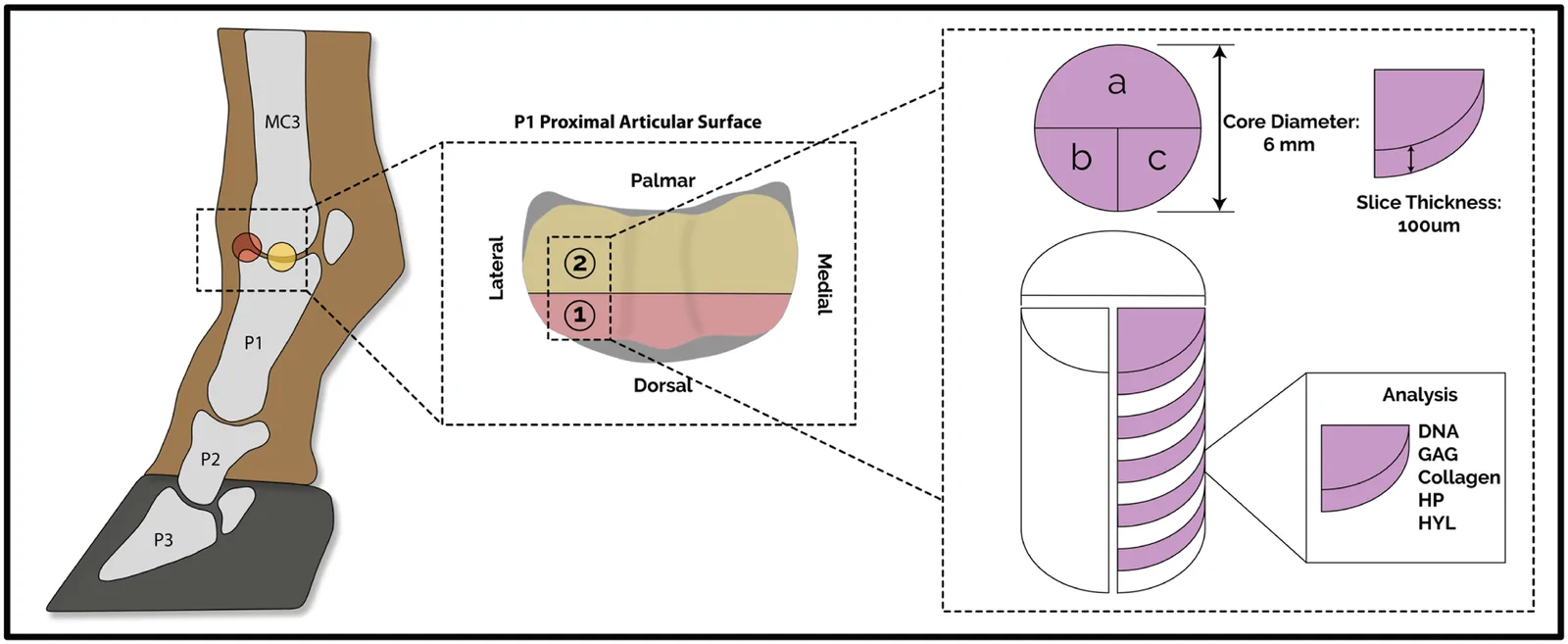
Schematic representation of sample harvest location from the dorsolateral (1) and central-lateral (2) aspect of proximal P1 with subsequent sectioning of the 6 mm core into 3 pieces before further sectioning of one “quarter” into 100um thick slices for biochemical analysis

### 2.3. Sample Analysis

#### 2.3.1. Biochemical Analysis

Sample analysis was performed across the full cartilage thickness of the sample with the cartilage sectioned into 100 μm cartilage slices using a microcryotome until the subchondral bone was reached.

#### 2.3.2. Quantification of Glycosaminoglycan & DNA Content

Cartilage samples were digested using papain (1 u/ml, Sigma P3125) in 400 μl of 50 mmol/l phosphate buffer (pH 6.5), containing 2 mmol/l Na_2_EDTA and 2 mmol/l cysteine for 4 h, at 65°C. A dimethylmethylene blue (DMMB) colorimetric assay was used to measure the polysulphated GAG content.^28^ Shark chondroitin sulphate was used as a standard. DNA content was measured using the protocol report by Kim et al.^29^ Hoechst 33258 was added to 80 μl of the papain-digested cartilage and fluorescence was measured immediately after mixing using a fluorimeter, with excitation at 365 nm and emission at 460 nm. Calf thymus DNA (Sigma D-4764) was used as a reference.

#### 2.3.3. Quantification of Total Collagen, Hydroxylysine (HYL) Content and Hydroxylysylpyridinoline (HP) Crosslinks

Papain digested samples were then hydrolyzed with 6M HCl at 110°C for 20h. The amount of hydroxyproline and proline was determined by reverse-phase high performance liquid chromatography (HPLC) of 9-fluorenylmethyl chloroformate (Fluka, Buchs, Switzerland)-derivatized amino acids, as described by Bank et al.^30^ Collagen content was calculated assuming 300 residues hydroxyproline per triple helix and a molecular weight of 300,000 g/mol. For the determination of the collagen cross-link hydroxylysylpyridinoline, acid-hydrolyzed samples were diluted to 50% acetic acid and injected on an HPLC system equipped with online sample purification on CC31 cellulose using a Prospekt solid-phase extractor (Separations, Jasco Benelux BV, IJsselstein, The Netherlands). The retained cross-links were eluted from the CC31 material and online chromatographed on a cation exchange column (Whatman Partisil SCX; Fisher Scientific, Waltham, MA). Eluting cross-links were detected by a Jasco fluorometer (model FP-920; Separations). The PYD/DPD HPLC calibrator (Metra, Palo Alto, CA) was used as standard. Values are expressed as total amount of residues per collagen molecule.^19,26,30-32^

### 2.4. Structural Analysis

#### 2.4.1. Parallelism and Orientation Using Quantitative Polarized Light Microscopy (qPLM)

Collagen fibril architecture was quantified using qPLM as previously reported.^23,33^ The Parallelism Index (PI) was determined, which assesses the degree of alignment of the collagen fibrils in relation to each other, ranging from 0% (random organization) to 100% (perfect parallelism). The orientation angle was also calculated and expressed in degrees, this angle quantifies the direction of the fibrils in relation to the articular surface, where 0^o^ is parallel to the articular surface and 90^o^ is perpendicular. Measurements were obtained across the full cartilage thickness.

### 2.5. Statistical Analysis

A generalized linear mixed-effects model was used with horse as a random effect and regimen (box or trained), slice depth (superficial, middle, deep), site (1 or 2), and their interactions as fixed effects. Model assumptions were assessed using simulation-based residual diagnostics (DHARMa), supported by descriptive statistics (mean, median, skewness), graphical assessment (histograms, density plots, QQ plots, residual vs. fitted plots), and statistical tests (e.g., Shapiro-Wilk) where applicable. Tukey was used for pairwise comparisons of estimated marginal means. Slice depth was treated as a categorical factor with three groups based on relative cartilage thickness: Superficial (0–20%), Middle (21–60%), and Deep (61–100%). All statistical tests were interpreted using a 5% level of significance (p<0.05). Depth-wise line plots were generated by normalizing the full slice thicknesses using a percentage of the total cartilage thickness to account for variations in slice number. All statistical analyses and plot generation were performed using R statistical programming language (R Core, 2023). All data were included in the analysis unless specifically noted.

## 3. Results

The total number of slices obtained ranged from 7 to 10 at site 1, and 6 to 10 at site 2 with the mean number of 9.38±1.02 and 8.56±1.31 respectively. Generalized linear mixed-effects modelling identified significant main effects of site, slice depth and/or regimen, no significant two- or three-way interactions were detected for GAG, DNA, collagen or HP. Glycosaminoglycan concentrations (Figure 3) were consistently higher at site 2 compared with site 1 across all slice depths and both exercise regimens (*p* < 0.001). Furthermore, within site 1, trained horses had higher GAG concentrations than box horses in the superficial (p = 0.004) and deep (p = 0.008) groups. Site 1 DNA content (Figure 4) exceeded site 2 across all slice groups and regimens (p<0.001) except for the superficial group in the trained regimen where there was no difference (p = 0.115). In the deep layer, total collagen content (Figure 5) was higher at site 1 for both regimens (box: *p* = 0.038; trained: *p*=0.007) and in the middle layer for the trained regimen only (p=0.004). Hydroxylysylpyridinoline (HP) content (Figure 6) showed consistent site differences, with significantly higher values at site 2 across all cartilage depths and exercise regimens (p < 0.004). Within-site comparisons revealed higher HP levels at site 1 in the superficial layer (p = 0.021) and at site 2 in the middle layer (p = 0.047) of the box horses only. Hydroxylysine (HYL) (Figure 7) showed significantly higher values at site 2 than site 1 in the superficial layer and middle layers of the trained group (p <0.001, p = 0.031). A significant two-way interaction between regimen and site was detected (p = 0.016). Orientation angle (Oa) (Figure 8) was significantly higher in the superficial group at site 1 compared to site 2 in both the box (p < 0.001) and trained (p = 0.001) groups. In the deep layers site 2 showed significantly higher values than site 1 (p = 0.007) in the trained group only. Significant two-way interactions were detected between regimen and site (p = 0.025) and slice depth and site (p < 0.001). Parallelism (PI) (Figure 9) was significantly higher in site 2 across all slice groups (p < 0.04) except for the middle group in the trained horses where no significance was detected. A regimen effect for PI was detected at site 1 only in the superficial (p = 0.008) and middle (p = 0.003) groups with trained being significantly higher than box. Significant 2-way interactions were detected between regimen and site (p = 0.003) and slice depth and site (p = 0.003). The results are summarised in Table 1 where the mean and median values are provided by site and regimen.

**Figure 3.**
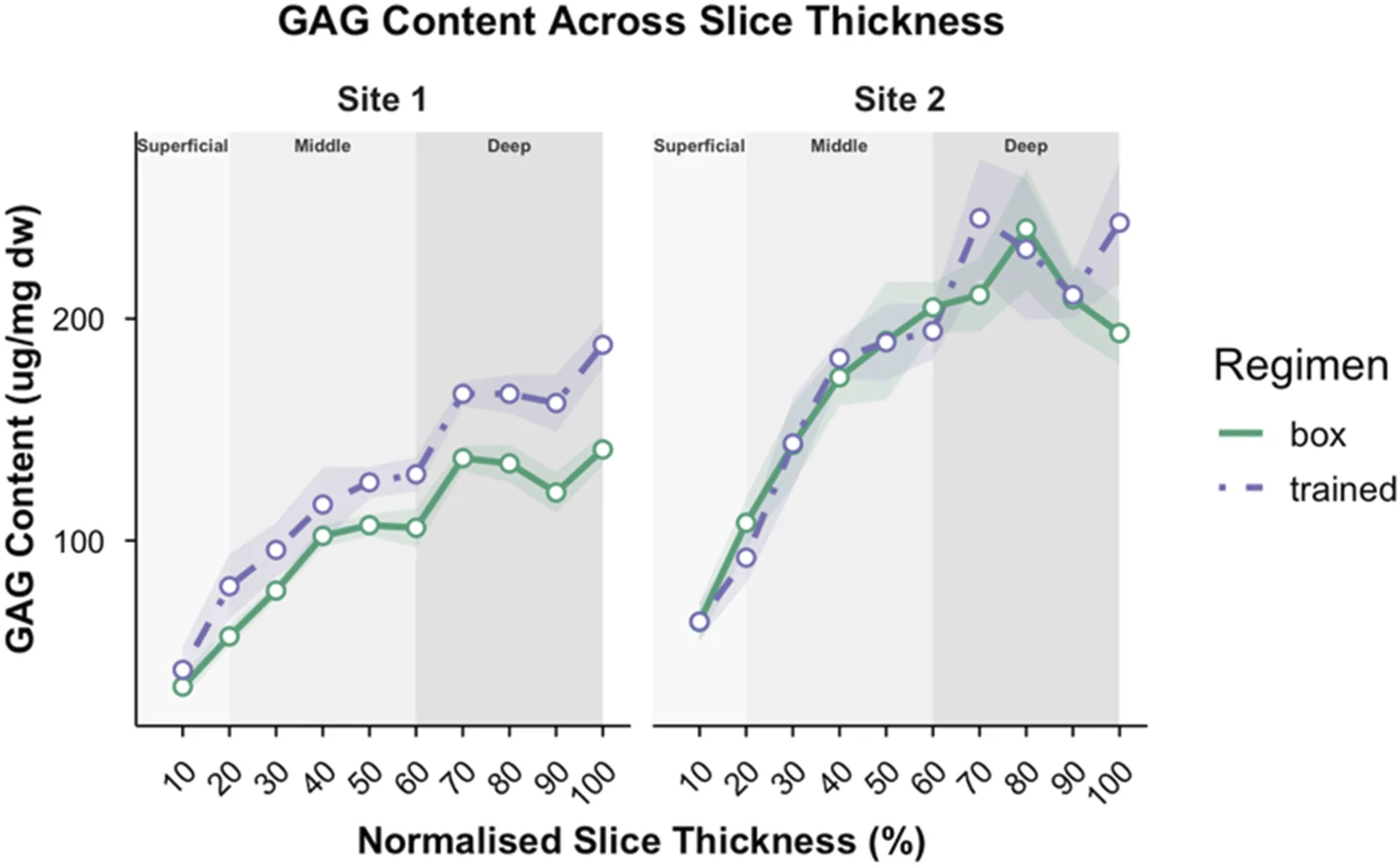
Line plot showing GAG content (mean±SE) across full slice thickness for each regimen between sites

**Figure 4.**
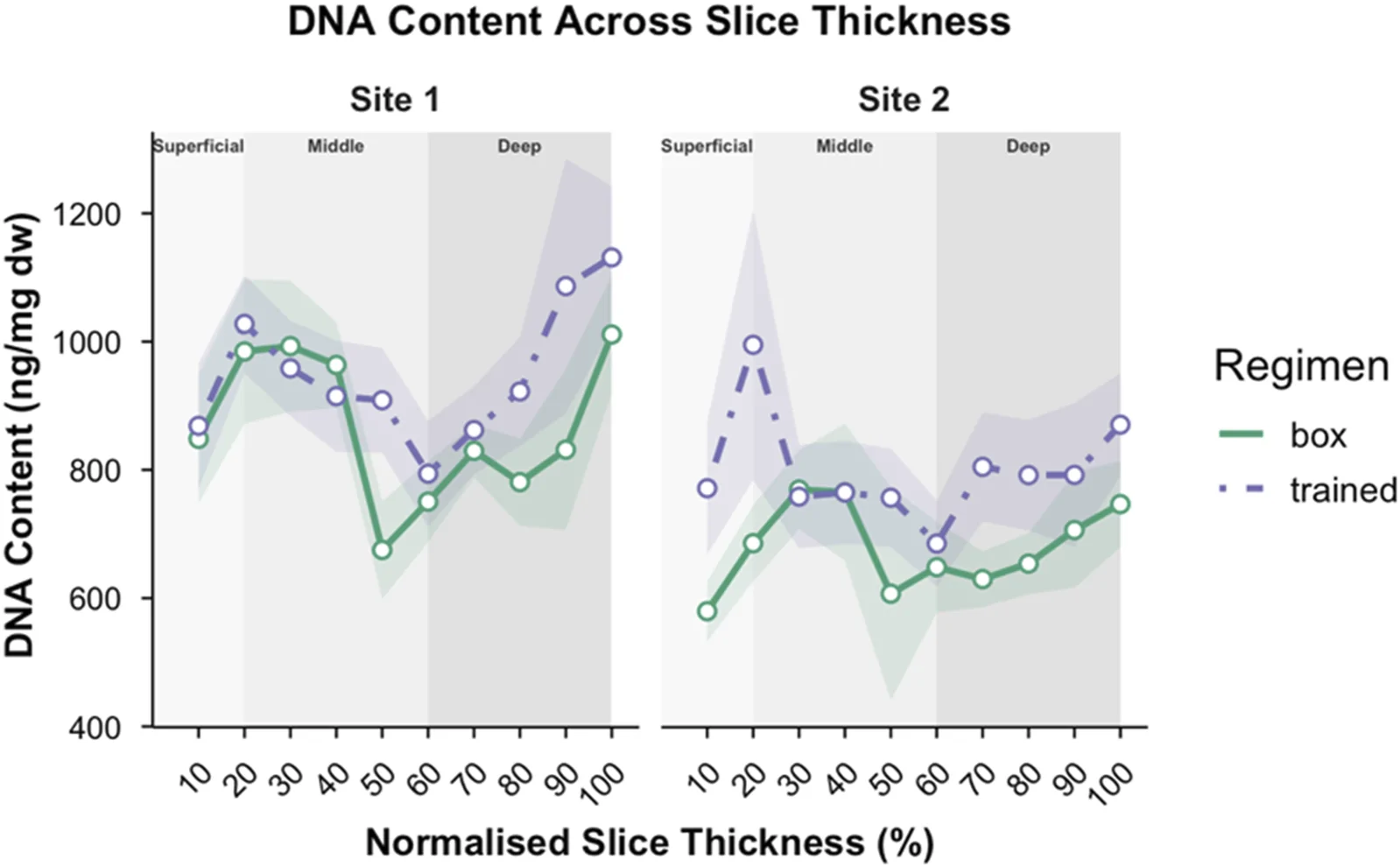
Line plot showing DNA content (mean±SE) across full slice thickness for each regimen between sites

**Figure 5.**
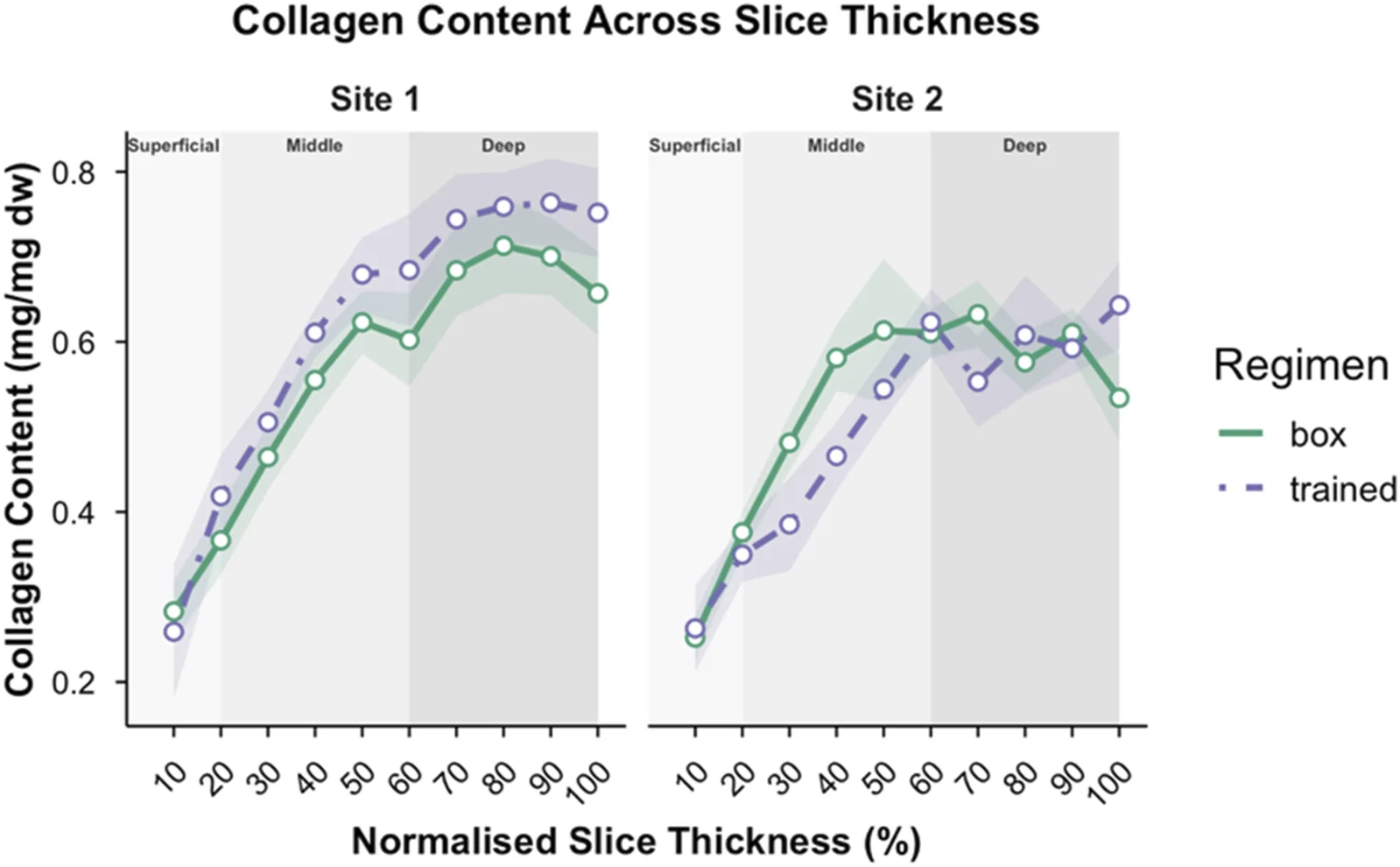
Line plot showing Collagen content (mean±SE) across full slice thickness for each regimen between sites

**Figure 6.**
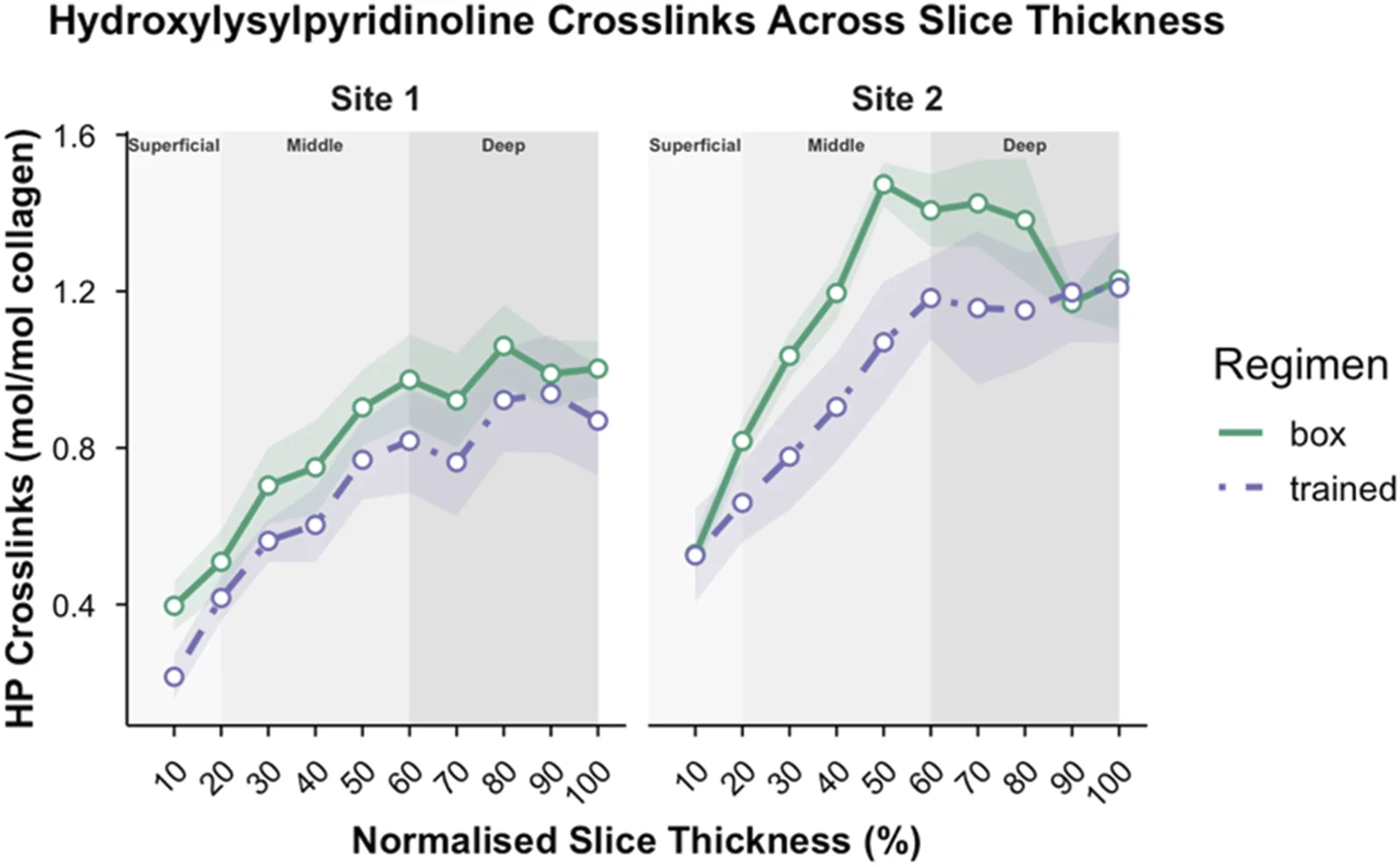
Line plot showing Hydroxylysylpyridinoline crosslinking (mean±SE) across full slice thickness for each regimen between sites

**Figure 7.**
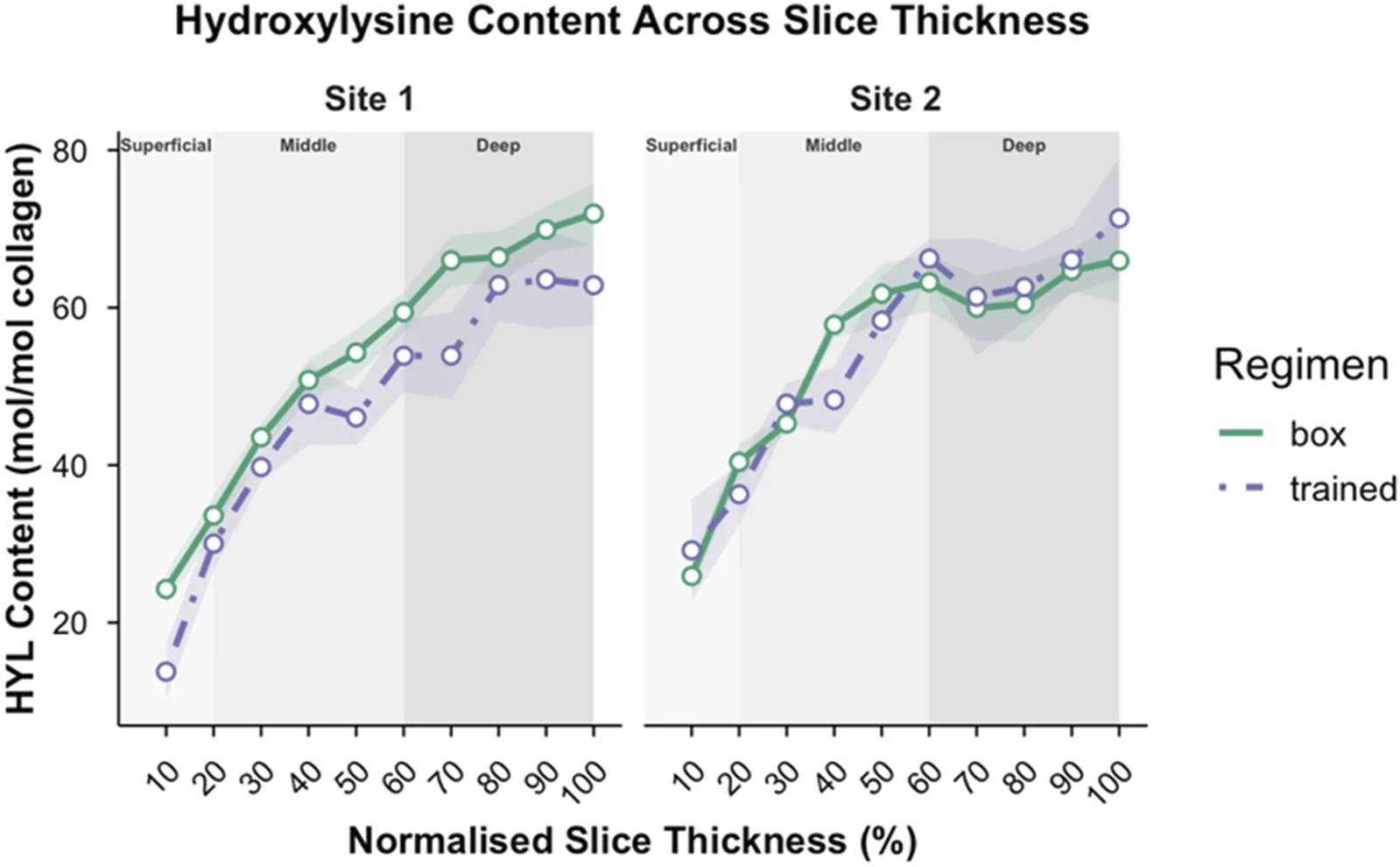
Line plot showing Hydroxylysine content (mean±SE) across full slice thickness for each regimen between sites

**Figure 8.**
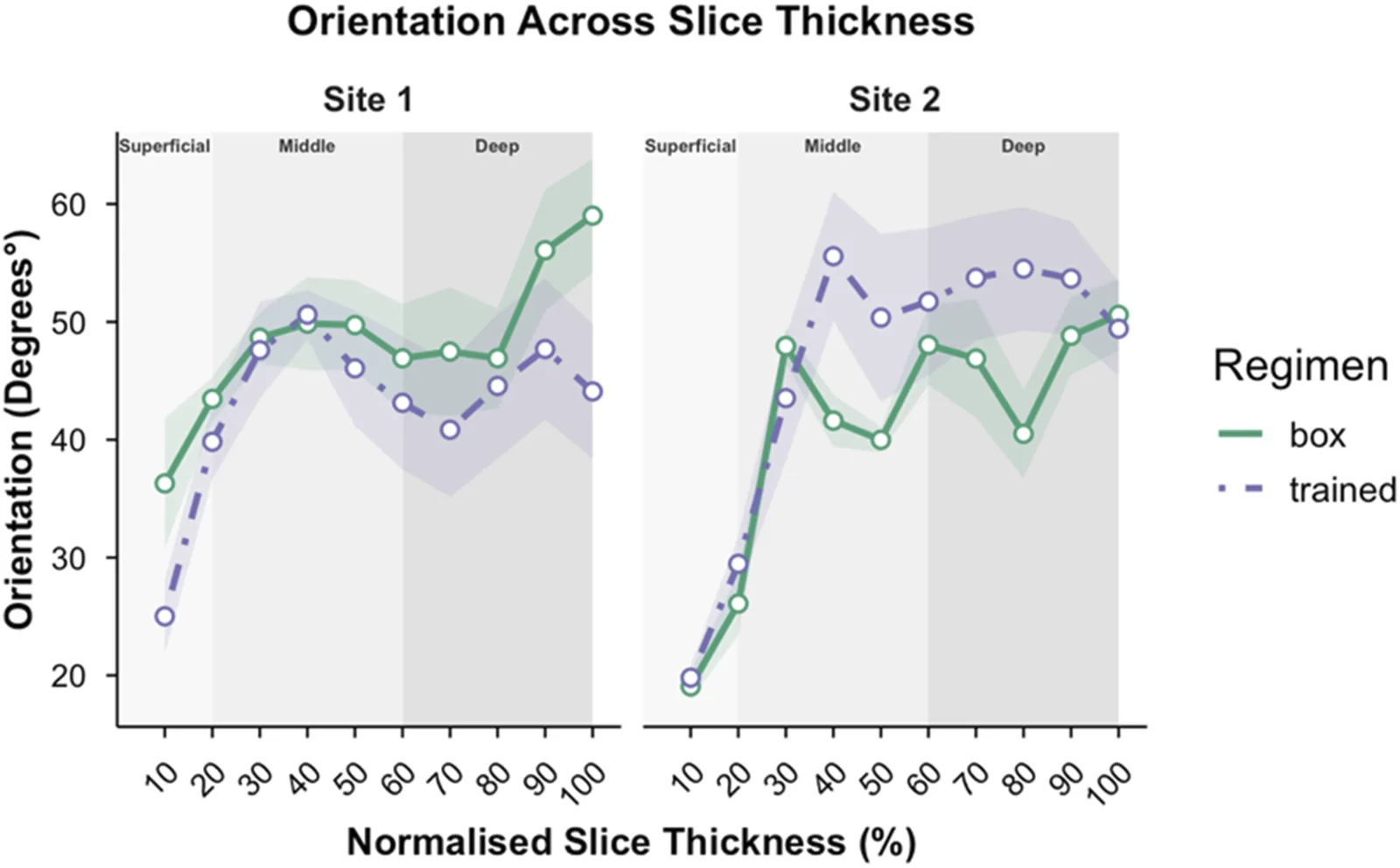
Line plot showing collagen fibril orientation angle (mean±SE) across full slice thickness for each regimen between sites

**Figure 9.**
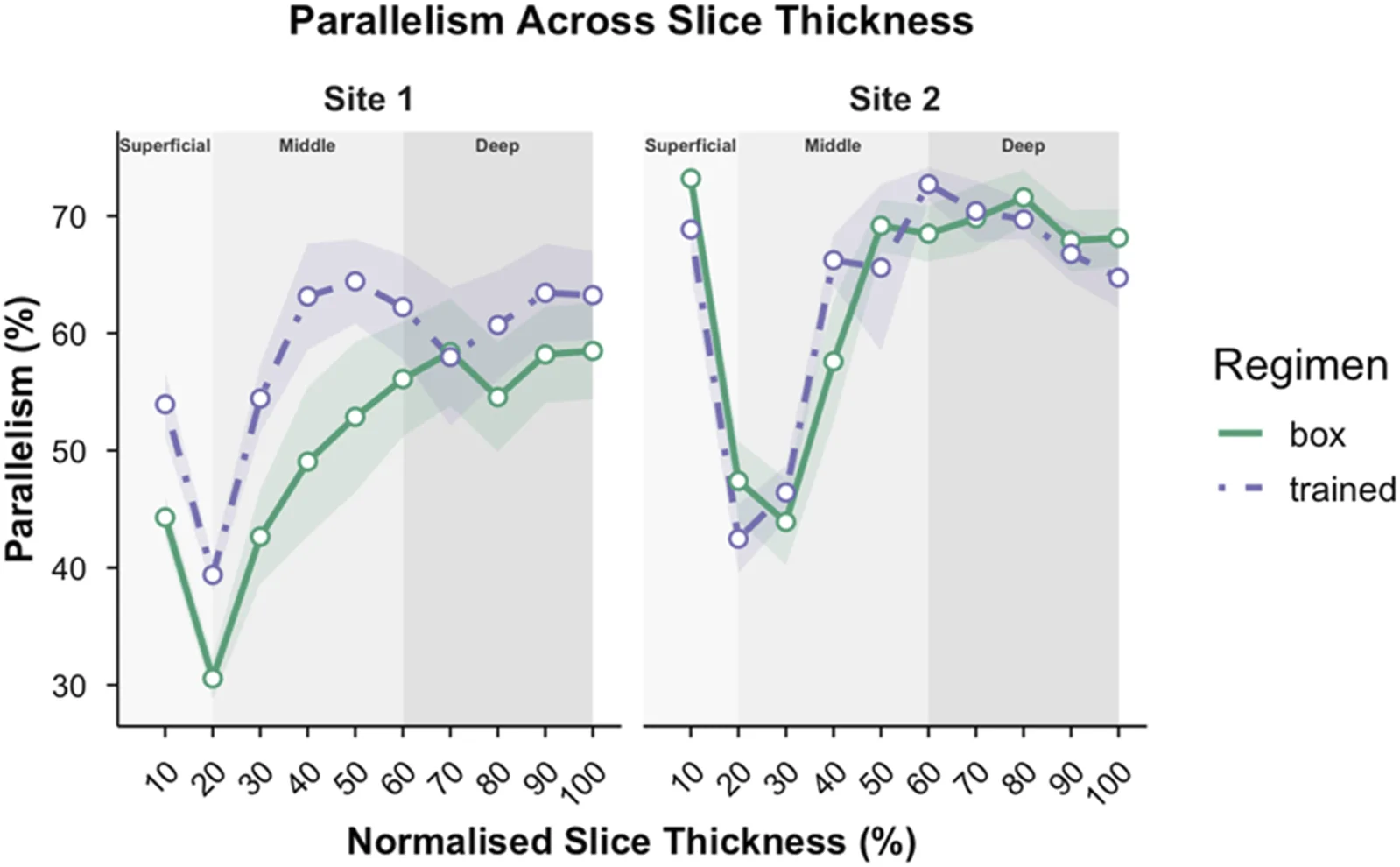
Line plot showing collagen fibril parallelism (mean±SE) across full slice thickness for each regimen between sites

**Table 1.** Summary of Overall Biochemical (Mean and Median) and Structural Analysis at Each Site Between the Two Regimens (Box and Trained)

Biochemical Analyte	Site 1				Site 2			
	Box		Trained		Box		Trained	
	Mean ± SD	Median ± IQR	Mean ± SD	Median ± IQR	Mean ± SD	Median ± IQR	Mean ± SD	Median ± IQR
Collagen (mg/mg dw)	0.56 (±0.08)	0.60 (±0.22)	0.62 (±0.04)	0.67 (±0.20)	0.52 (±0.05)	0.55 (±0.15)	0.50 (±0.05)	0.54 (±0.14)
DNA (ng/mg dw)	871.67 (±127.28)	862.55 (±146.25)	957.61 (±120.39)	959.84 (±147.33)	679.57 (±135.56)	681.05 (±141.49)	816.49 (±201.02)	789.40 (±130.71)
GAG (µg/mg dw)	101.25 (±7.23)^c^	110.36 (±49.17)	126.71 (±12.77)	130.20 (±67.65)	171.32 (±21.84)	187.92 (±60.44)	176.46 (±25.35)	183.98 (±105.26)
HP (mol/mol collagen)	0.82 (±0.24)	0.94 (±0.34)	0.70 (±0.25)	0.75 (±0.27)	1.13 (±0.09)	1.17 (±0.33)	0.98 (±0.25)	1.13 (±0.28)
HYL (mol/mol collagen)	53.97 (±2.54)	56.44 (±21.85)	47.58 (±7.48)	52.61 (±16.92)	54.14 (±1.82)	57.67 (±19.18)	54.76 (±4.80)	60.24 (±17.19)
Oa (degrees)	46.63 (±7.56)	47.92 (±9.21)	42.71 (±10.71)	47.21 (±10.22)	41.18 (±4.31)	44.15 (±10.89)	44.33 (±10.90)	47.82 (±12.99)
PI (percent)	50.32 (±8.55)	51.41 (±10.19)	58.29 (±7.76)	60.33 (±8.83)	63.62 (±3.97)	67.86 (±9.62)	62.99 (±3.94)	69.27 (±9.42)

### 4. Discussion

The biochemical composition of cartilage at sites 1 and 2 differed significantly in at least one regimen-site combination for all biochemical and structural components analysed. It has been previously established that the two sites experience different loading conditions at rest and exercise.^27^ Sectioning the cartilage into 100 μm layers, rather than analysing full-thickness samples, enabled depth-specific biochemical assessment and accounted for the gradual compositional changes across the cartilage depth providing valuable and relatively unique insight.

The consistently higher GAG content at site 2 for both the box and trained horses is consistent with other reports showing the same finding between horses kept at pasture compared with the same training regimen used in this study.^20^ It was shown in the study reported here that GAG content increased in both sites and exercise regimens from superficial to deep which is again consistent with previous reports in horses^20^ and other species.^13,34-36^ The effects of training were evident at site 1 with significantly increased GAG content in the superficial and deep zones when compared to the horses maintained on box rest. This supports the findings of Saadat et al, who found an increase in proteoglycan content in the deep zone associated with increased loading in a rabbit model.^37^ The optimal exercise loading has not yet been determined but light and moderate exercise in rodents caused an increase in femoral condyle cartilage GAG content compared to a sedentary control whereas high intensity caused a reduction.^38^ In dogs, moderate exercise increased GAG content by 28% in the femoral condyles in the intermediate and deep zones but not in the superficial zones.^39^ In treadmill-trained young Thoroughbreds, Murray et al (2001) found an increase in GAG-content in the cartilage from the dorsal radial and dorsal intermediate carpal bone surfaces, which are sites known to be heavily loaded during exercise, like the dorsal margin of the first phalanx examined in our study.^40^ The data from the Bristol study were corroborated by Bird et al (2000) who showed that proteoglycan synthesis rates were higher in the trained animals than in the controls.^41^

The collagen distribution follows a similar pattern to that of GAG with increasing levels from superficial to deep which is contrary to Fourier Transform Infrared (FTIR) spectroscopy reports in older more mature cartilage which show an apparent inverse relationship.^12,20^ However, FTIR presents challenges for quantification of collagen and proteoglycan content due to significant amide band overlapping which can introduce significant errors and, particularly when analysed by univariate methods which have been widely used, should be interpreted with caution.^12,42^ Collagen distribution does appear to be subject to changes in spatial distribution with increasing age in pigs.^23^ Site 1 collagen content was higher in the deeper layers compared to site 2 in both box and trained horses which supports similar findings in adult horses where the dorsal and palmar aspects of the proximal first phalanx contain greater collagen than the central portion.^22,24,26^ Interestingly, there was also an increase in the middle layers at site 1 of the trained group which may be the result of increased loading at this site which is maximally loaded only during exercise.^27^ This increase in collagen at site 1, which is subjected to approximately 30%-50% greater maximal load during exercise than site 2,^22,27^ is likely the result of functional adaptation. Differences in mechanical loading at these sites are detected by chondrocytes and through mechanotransduction pathways influence chondrocyte proliferation, collagen/proteoglycan synthesis and matrix organisation.^43-45^ Increases in collagen content of ∼35% have been reported at site 1 compared with only ∼10% at site 2 during the first 5 months of life when foal cartilage is experiencing a rapid transition from total lack of compressive loading *in utero* to full compressive and shear loading during weightbearing and exercise with topographical variations.^18^ Functional adaptation is the normal collagen maturation process by which biochemical and biomechanical heterogeneity develop and is considered critical for the development of healthy cartilage able to withstand the adult forces exerted upon it whilst resisting injury. This is of particular importance, given the fact that mature cartilage has very limited reparative capacity.^46^

The depth- and site-dependent variations in collagen content were accompanied by corresponding differences in HYL and HP, reflecting distinct stages of collagen network synthesis and maturation. Hydroxylysine is a post-translationally modified amino acid unique to collagen, formed by hydroxylation of lysine residues during collagen biosynthesis, and reflects hydroxylation activity and new collagen formation.^47^ Hydroxylysylpyridinoline is a non-reducible cross-link formed between hydroxylysine residues during extracellular collagen maturation and reflects collagen network stabilization and tissue maturity.^48^ Collagen concentrations increased with depth and were significantly higher at site 1 in the deeper layers, whereas both HYL and HP were increased at site 2 in specific depth/regimen combinations, indicating a more actively remodelled yet biochemically mature matrix at this location.

The higher HYL values at site 2 in the superficial and middle trained group suggest greater hydroxylation activity and collagen synthesis, consistent with ongoing matrix formation preceding cross-link maturation and supports previous work.^19^ In contrast, consistently higher HP levels at site 2 across all depths indicate a more extensively cross-linked and stable collagen network. Previous work by this group found there was no statistically significant difference between site 1 and site 2 at 5 months when samples were harvested from the medial aspect of the joint^18,19^ and in adult horses, site 1 tends to be higher.^19,24,49^ Together, these findings suggest that site 2 exhibits both active collagen remodelling and advanced matrix maturation, whereas site 1 shows higher collagen accumulation but comparatively lower cross-link density. Loading is key to this maturation process. Site 2 is consistently loaded at all stages from birth, but site 1 is only subjected to increasing load when exercised and therefore maturation is slower to develop at this site. It is likely given the higher clinical prevalence of lateral metacarpal condylar fractures^50-52^ that there are differences between the loading of the medial and lateral aspects of the joint which may contribute to these findings but this has not been proven.^24^ However, it has been demonstrated that cartilage degeneration starts at the dorsomedial aspect of P1 and extends to the dorsolateral aspect with further degeneration propagating from the medial to lateral fovea.^53^ Overall, these results highlight a dynamic interplay between collagen synthesis, cross-link maturation, and total collagen content, governed by site-specific mechanical loading and exercise history.

Collagen fibril orientation was not associated with any exercise related differences as a main effect, but distinct site-dependent patterns were evident. In the superficial zone, site 1 exhibited higher Oa values than site 2, indicating a less horizontally aligned collagen network. In contrast, within the deep zone the Oa was higher in the trained group at site 2 compared to site 1, reflecting a more vertically oriented fibrillar structure. The differences in orientation can be explained by the differences in loading at site 1 compared with site 2 and the additional load provided by training and highlights the apparently accelerating effect loading has on the development of the final architecture as originally described by Benninghoff (1925).^2^ A similar observation was made in Thoroughbred yearlings subjected to another equine exercise study^54^ in which a similar depth-wise analysis of the articular cartilage was performed.^22^ Equine cartilage at 5 months in this study appears to be more advanced in its development compared to porcine at 4 months^23^ where collagen fibril orientation was predominantly parallel to the articular surface. This may represent differences in loading of the joints of the extremities in juvenile pigs compared with foals during the early stages of life particularly related to their housing and management, which emphasises the importance of the early life stages in cartilage development.

Parallelism, which reflects the degree of fibril alignment relative to each other, was generally higher at site 2 across most depth zones, indicating a more organized collagen network under consistent load. Localized exercise effects were observed at site 1 in the superficial and middle zones, where trained horses showed significantly higher PI than the box group, suggesting that training can enhance collagen fibril alignment in more superficially loaded regions. This data supports those reported previously from the medial aspect of the same joint^33^ and adds new insight in regard to the effect of training. In summary, the structural data (Oa and PI) would suggest that collagen organization is both site- and depth-dependent, with mechanical load influential on functional adaptation of the cartilage matrix.

Only two sites were examined in this study, which limits the extent to which the observed differences can be generalised across the associated articular surfaces. Future work should therefore assess topographical variation across a greater area of the articular surface. The primary experimental comparison of this study was between training regimens, for which differences can be directly attributed to the imposed training conditions. Zonal differences whilst observed are only indirectly related to local loading conditions. Zonal variation should therefore be considered a secondary outcome and interpreted cautiously. Whilst the sample size was relatively small with 8 horse per group, the numbers are in keeping with similar studies of this nature. The harvested cores were sectioned to allow the use of multiple analytical methods as shown in Figure 2 maximising the analytic potential of the samples.

In conclusion, this study illustrates the site differences in biochemical and structural components of equine cartilage at 5 months under different training regimes. The more central consistently loaded Site 2 exhibited higher GAG and HP content with HYL higher in the superficial and middle layers of the trained group, while the during exercise intermittently loaded dorsal site 1 showed greater collagen accumulation in deeper layers but lower cross-link density. Structural analyses (Oa and PI) confirmed that collagen fibril orientation and alignment adapt regionally, with training enhancing fibril organization in superficial zones. Together, these findings highlight the dynamic interplay between collagen synthesis, maturation, and fibril organization, demonstrating that mechanical load is a key driver of cartilage biochemical composition and structure essential for functional cartilage adaptation.

## References

[1] Buckwalter JA Mankin HJ . Articular cartilage: Part I. J Bone Joint Surg Am. 1997;79(4):600.

[2] Benninghoff A . Form und Bau der Gelenkknorpel in ihren Beziehungen zur Funktion. Z Für Anat Entwicklungsgeschichte. 1925;76(1):43-63. doi:10.1007/BF02134417.

[3] Mow VC Ratcliffe A Robin Poole A . Cartilage and diarthrodial joints as paradigms for hierarchical materials and structures. Biomaterials. 1992;13(2):67-97. doi:10.1016/0142-9612(92)90001-5. 1550898

[4] Hunziker EB Michel M Studer D . Ultrastructure of adult human articular cartilage matrix after cryotechnical processing. Microsc Res Tech. 1997;37(4):271-284. 10.1002/(SICI)1097-0029(19970515)37:4<271::AID-JEMT3>3.0.CO;2-O 9185150

[5] Hunziker EB Quinn TM Häuselmann HJ . Quantitative structural organization of normal adult human articular cartilage. Osteoarthritis Cartilage. 2002;10(7):564-572. 10.1053/joca.2002.0814 12127837

[6] Poole CA Flint MH Beaumont BW . Morphological and functional interrelationships of articular cartilage matrices. J Anat. 1984;138(Pt 1):113-138. PMC1164314 6706831

[7] Clark JM . The organization of collagen in cryofractured rabbit articular cartilage: A scanning electron microscopic study. J Orthop Res. 1985;3(1):17-29. doi:10.1002/jor.1100030102. 3981292

[8] Wong M Wuethrich P Eggli P Hunziker E . Zone‐specific cell biosynthetic activity in mature bovine articular cartilage: A new method using confocal microscopic stereology and quantitative autoradiography. J Orthop Res. 1996;14(3):424-432. doi:10.1002/jor.1100140313. 8676256

[9] Karpiński R Prus A Baj J , et al. Articular Cartilage: Structure, Biomechanics, and the Potential of Conventional and Advanced Diagnostics. Appl Sci. 2025;15(12):6896. doi:10.3390/app15126896.

[10] Aspden RM Hukins DWL . Collagen organization in articular cartilage, determined by X-ray diffraction, and its relationship to tissue function. Proc R Soc Lond B Biol Sci. 1981;212(1188):299-304. doi:10.1098/rspb.1981.0040. 6115394

[11] Ratcliffe A Fryer PR Hardingham TE . The distribution of aggregating proteoglycans in articular cartilage: comparison of quantitative immunoelectron microscopy with radioimmunoassay and biochemical analysis. J Histochem Cytochem. 1984;32(2):193-201. doi:10.1177/32.2.6363519. 6363519

[12] Yin J Xia Y Lu M . Concentration Profiles of Collagen and Proteoglycan in Articular Cartilage by Fourier Transform Infrared Imaging and Principal Component Regression. Spectrochim Acta A Mol Biomol Spectrosc. 2012;88:90-96. doi:10.1016/j.saa.2011.12.002. PMC3268907 22197357

[13] Sophia Fox AJ Bedi A Rodeo SA . The Basic Science of Articular Cartilage: Structure, Composition, and Function. Sports Health Multidiscip Approach. 2009;1(6):461-468. doi:10.1177/1941738109350438. PMC3445147 23015907

[14] Mansour JM . Biomechanics of Cartilage. Eng Biol. 2013. Published online.

[15] Redler I Mow VC Zimny ML Mansell J . The ultrastructure and biomechanical significance of the tidemark of articular cartilage. Clin Orthop. 1975;112:357-362. 1192647

[16] Maroudas A . Balance between swelling pressure and collagen tension in normal and degenerate cartilage. Nature. 1976;260(5554):808-809. doi:10.1038/260808a0. 1264261

[17] Långsjö TK Hyttinen M Pelttari A Kiraly K Arokoski J Helminen HJ . Electron microscopic stereological study of collagen fibrils in bovine articular cartilage: volume and surface densities are best obtained indirectly (from length densities and diameters) using isotropic uniform random sampling. J Anat. 1999;195(Pt 2):281-293. doi:10.1046/j.1469-7580.1999.19520281.x. PMC1467991 10529063

[18] Brama PAJ Tekoppele JM Bank RA Barneveld A Van Weeren PR . Functional adaptation of equine articular cartilage: the formation of regional biochemical characteristics up to age one year. Equine Vet J. 2000;32(3):217-221. doi:10.2746/042516400776563626. 10836476

[19] Brama PAJ TeKoppele JM Bank RA Barneveld A van Weeren PR . Development of biochemical heterogeneity of articular cartilage: influences of age and exercise. Equine Vet J. 2002;34(3):265-269. doi:10.2746/042516402776186146. 12108744

[20] Brama PAJ Holopainen J Van Weeren PR Firth EC Helminen HJ Hyttinen MM . Influence of exercise and joint topography on depth-related spatial distribution of proteoglycan and collagen content in immature equine articular cartilage. Equine Vet J. 2009;41(6):557-563. doi:10.2746/042516409X424162. 19803051

[21] Brommer H Brama Pa. J Laasanen MS Helminen HJ van Weeren PR Jurvelin JS . Functional adaptation of articular cartilage from birth to maturity under the influence of loading: a biomechanical analysis. Equine Vet J. 2005;37(2):148-154. doi:10.2746/0425164054223769. 15779628

[22] van Weeren PR Firth EC Brommer H , et al. Early exercise advances the maturation of glycosaminoglycans and collagen in the extracellular matrix of articular cartilage in the horse. Equine Vet J. 2008;40(2):128-135. doi:10.2746/042516408X253091. 18093892

[23] Rieppo J Hyttinen MM Halmesmaki E , et al. Changes in spatial collagen content and collagen network architecture in porcine articular cartilage during growth and maturation. Osteoarthritis Cartilage. 2009;17(4):448-455. doi:10.1016/j.joca.2008.09.004. 18849174

[24] Brama PAJ Tekoppele JM Bank RA Karssenberg D Barneveld A Van Weeren PR . Topographical mapping of biochemical properties of articular cartilage in the equine fetlock joint. Equine Vet J. 2000;32(1):19-26. doi:10.2746/042516400777612062. 10661380

[25] Van Weeren PR Barneveld A . Introduction: Study design to evaluate the influence of exercise on the development of the musculoskeletal system of foals up to age 11 months. Equine Vet J. 1999;31(S31):4-8. doi:10.1111/j.2042-3306.1999.tb05307.x. 10999654

[26] Brama PAJ TeKoppele JM Bank RA van Weeren PR Barneveld A . Influence of site and age on biochemical characteristics of the collagen network of equine articular cartilage. Am J Vet Res. 1999;60(3):341-345. 10188817

[27] Brama Pa. J Karssenberg D Barneveld A van Weeren PR . Contact areas and pressure distribution on the proximal articular surface of the proximal phalanx under sagittal plane loading. Equine Vet J. 2001;33(1):26-32. doi:10.2746/042516401776767377. 11191606

[28] Farndale RW Buttle DJ Barrett AJ . Improved quantitation and discrimination of sulphated glycosaminoglycans by use of dimethylmethylene blue. Biochim Biophys Acta. 1986;883(2):173-177. doi:10.1016/0304-4165(86)90306-5. 3091074

[29] Kim YJ Sah RLY Doong JYH Grodzinsky AJ . Fluorometric assay of DNA in cartilage explants using Hoechst 33258. Anal Biochem. 1988;174(1):168-176. doi:10.1016/0003-2697(88)90532-5. 2464289

[30] Bank RA Beekman B Verzijl N de Roos JA Sakkee AN TeKoppele JM . Sensitive fluorimetric quantitation of pyridinium and pentosidine crosslinks in biological samples in a single high-performance liquid chromatographic run. J Chromatogr B Biomed Sci App. 1997;703(1-2):37-44. doi:10.1016/s0378-4347(97)00391-5. 9448060

[31] Bank RA Jansen EJ Beekman B te Koppele JM . Amino Acid Analysis by Reverse-Phase High-Performance Liquid Chromatography: Improved Derivatization and Detection Conditions with 9-Fluorenylmethyl Chloroformate. Anal Biochem. 1996;240(2):167-176. doi:10.1006/abio.1996.0346. 8811901

[32] Bank RA Bayliss MT Lafeber FP Maroudas A Tekoppele JM . Ageing and zonal variation in post-translational modification of collagen in normal human articular cartilage. The age-related increase in non-enzymatic glycation affects biomechanical properties of cartilage. Biochem J. 1998;330(Pt 1):345-351. doi:10.1042/bj3300345. PMC1219146 9461529

[33] Hyttinen MM Holopainen J René van Weeren P Firth EC Helminen HJ Brama PAJ . Changes in collagen fibril network organization and proteoglycan distribution in equine articular cartilage during maturation and growth. J Anat. 2009;215(5):584-591. doi:10.1111/j.1469-7580.2009.01140.x. PMC2780575 19732210

[34] Camacho NP West P Torzilli PA Mendelsohn R . FTIR microscopic imaging of collagen and proteoglycan in bovine cartilage. Biopolymers. 2001;62(1):1-8. doi:10.1002/1097-0282(2001)62:1<1::AID-BIP10>3.0.CO;2-O. 11135186

[35] Rieppo L Rieppo J Jurvelin JS Saarakkala S . Fourier Transform Infrared Spectroscopic Imaging and Multivariate Regression for Prediction of Proteoglycan Content of Articular Cartilage. PLOS ONE. 2012;7(2):e32344. doi:10.1371/journal.pone.0032344. PMC3281137 22359683

[36] Arokoski MEA Tiitu V Jurvelin JS Korhonen RK Fick JM . Topographical investigation of changes in depth-wise proteoglycan distribution in rabbit femoral articular cartilage at 4 weeks after transection of the anterior cruciate ligament. J Orthop Res. 2015;33(9):1278-1286. doi:10.1002/jor.22906. 25820864

[37] Saadat E Lan H Majumdar S Rempel DM King KB . Long-term cyclical in vivoloading increases cartilage proteoglycan content in a spatially specific manner: an infrared microspectroscopic imaging and polarized light microscopy study. Arthritis Res Ther. 2006;8(5):R147. doi:10.1186/ar2040. PMC1779443 16956418

[38] Ni GX Liu SY Lei L Li Z Zhou YZ Zhan LQ . Intensity-Dependent Effect of Treadmill Running on Knee Articular Cartilage in a Rat Model. BioMed Res Int. 2013;2013(1):172392. doi:10.1155/2013/172392. PMC3892754 24693534

[39] Kiviranta I Tammi M Jurvelin J Säämänen AM Helminen HJ . Moderate running exercise augments glycosaminoglycans and thickness of articular cartilage in the knee joint of young beagle dogs. J Orthop Res. 1988;6(2):188-195. doi:10.1002/jor.1100060205. 3278079

[40] Murray RC Birch HL Lakhani K Goodship AE . Biochemical composition of equine carpal articular cartilage is influenced by short-term exercise in a site-specific manner. Osteoarthritis Cartilage. 2001;9(7):625-632. doi:10.1053/joca.2001.0462. 11597175

[41] Bird JLE Platt D Wells T May SA Bayliss MT . Exercise‐induced changes in proteoglycan metabolism of equine articular cartilage. Equine Vet J. 2000;32(2):161-163. doi:10.2746/042516400777591624. 10743973

[42] Rieppo L Töyräs J Saarakkala S . Vibrational spectroscopy of articular cartilage. Appl Spectrosc Rev. 2017;52(3):249-266. doi:10.1080/05704928.2016.1226182.

[43] Jia Y Le H Wang X , et al. Double-edged role of mechanical stimuli and underlying mechanisms in cartilage tissue engineering. Front Bioeng Biotechnol. 2023;11:1271762. doi:10.3389/fbioe.2023.1271762. PMC10694366 38053849

[44] Wang N Lu Y Rothrauff BB , et al. Mechanotransduction pathways in articular chondrocytes and the emerging role of estrogen receptor-α. Bone Res. 2023;11(1):13. doi:10.1038/s41413-023-00248-x. PMC9984452 36869045

[45] Kacprzak B Stańczak M Bielenda B Yarmohammadi AA Hagner-Derengowska M . Molecular Aspects of Cartilage Microfracturation: Rehabilitation Insights. Orthop Rev. 2025;17:129917. doi:10.52965/001c.129917. PMC12021420 40276361

[46] Malda J Groll J Van Weeren PR . Rethinking articular cartilage regeneration based on a 250-year-old statement. Nat Rev Rheumatol. 2019;15(10):571-572. doi:10.1038/s41584-019-0278-7. 31367006

[47] Yamauchi M Sricholpech M . Lysine post-translational modifications of collagen. Essays Biochem. 2012;52:113-133. doi:10.1042/bse0520113. PMC3499978 22708567

[48] Açil Y Müller PK . Rapid method for the isolation of the mature collagen cross-links, hydroxylysylpyridinoline and lysylpyridinoline. J Chromatogr A. 1994;664(2):183-188. doi:10.1016/0021-9673(94)87006-3. 21941695

[49] Brama PAJ Tekoppele JM Bank RA van Weeren PR Barneveld A . Influence of different exercise levels and age on the biochemical characteristics of immature equine articular cartilage. Equine Vet J. 1999;31(S31):55-61. doi:10.1111/j.2042-3306.1999.tb05314.x. 10999661

[50] Zekas LJ Bramlage LR Embertson RM Hance SR . Characterisation of the type and location of fractures of the third metacarpal/metatarsal condyles in 135 horses in central Kentucky (1986-1994). Equine Vet J. 1999;31(4):304-308. doi:10.1111/j.2042-3306.1999.tb03821.x. 10454088

[51] Parkin TDH Proudman CJ French NP , et al. Risk of fatal distal limb fractures among thoroughbreds involved in the five tpes of racing in the United Kingdom. Vet Rec. 2010;154(16):493-497. doi:10.1136/vr.154.16.493. 15130054

[52] Jacklin BD Wright IM . Frequency distributions of 174 fractures of the distal condyles of the third metacarpal and metatarsal bones in 167 Thoroughbred racehorses (1999-2009). Equine Vet J. 2012;44(6):707-713. doi:10.1111/j.2042-3306.2012.00558.x. 22409440

[53] Brommer H Van Weeren PR Brama Pa. J Barneveld A . Quantification and age-related distribution of articular cartilage degeneration in the equine fetlock joint. Equine Vet J. 2003;35(7):697-701. doi:10.2746/042516403775696357. 14649362

[54] Rogers CW Firth EC McIlwraith CW , et al. Evaluation of a new strategy to modulate skeletal development in Thoroughbred performance horses by imposing track-based exercise during growth. Equine Vet J. 2008;40(2):111-118. doi:10.2746/042516408X268923. 18093894

